# Mr. Percivall's Elementary Lectures on the Veterinary Art

**Published:** 1823-12-01

**Authors:** 


*A Series of Elementary Lectures on the Veterinary Art; wherein the Anatomy, Physiology, and Pathology of the Horse are essayed on the General Principles of Medical Science.* By Veterinary Surgeon PERCIVALL, of the Royal Regiment of Artillery. Octavo, pp. 377. London, 1823.

To the practitioner in medicine, whether physician, surgeon, or surgeon-apothecary, the horse is an object of peculiar attention. This noblest of all animals is the medical man's attendant and devoted servant in all his rounds—transporting him from place to place, through sun and rain, by day and by night, with unexampled alacrity, patience, and expedition. That science, therefore, which has for its objects the preservation in health, and restoration from disease, of an animal nearest to man himself in the scale of animated nature, is particularly calculated to attract the serious regard of the faculty, and is far more closely allied to their own direct studies than many others which occupy much of their time and attention. The veterinary art is now patronised by Royalty itself, and immense savings in the wear and tear of the horse have been effected since the said art has got into the hands of men of science. In the system of shoeing alone, great advantages have been obtained. Many very formidable diseases of the foot are now warded off, by which horses are enabled to go with ease to themselves and safety to the rider. Formerly it was otherwise. When the board of ordnance first placed their horses under the superintendance of veterinary surgeons, numbers of these animals, highly valuable in other respects, "were cast for death purely because their feet were incurably diseased." Thrush, and its sequel canker, corns, grease, and all those varieties of disorganization induced by maltreated inflammation of the parts within the hoof, consigned multitudes of them to the knacker's knife.

"Whereas, since the eye of science has controled the hand of the smith, we will venture to assert, *that not a single horse has been lost to the service from either of the abovenamed diseases ; and that at the present day the three first*—viz. *thrush, canker, and corns, are almost unknown ; and the last of so rare occurrence, that there are not admitted into the Infirmary a dozen cases in the course of the year.*"—*Pref.* xii.

To the enlightened practitioners in medicine, veterinary researches may, from time to time, contribute information, on account of the extensive opportunities which veterinary surgeons enjoy in comparative anatomy and pathology.

"And where is the surgeon who would not himself direct the treatment of his sick or lame horse, in preference to calling in the village blacksmith?—a man whose knowledge is necessarily confined to that of nailing the shoe to the foot? He would not permit a shoemaker to visit one of his own patients, why should he allow the blacksmith to prescribe for his horse? The *theory* of medicine in the human subject, is the theory of medicine in the brute; it is the application of that theory—the *practice* alone that is different; whether we prescribe for a man, or a horse, a dog, or a cat, the laws of the animal economy are the same in all, and one, and that an unerring system of principles, built upon ascertained and established truths, is to dictate our practice in all." xiv.

It will not be expected that we should go into the elementary matter, of which the work is almost entirely composed ; but only glance at such points of theory or practice as may appear most interesting to those not professedly studying the subject.

1. *Blood.* In the first lecture, which is on the blood, we observe the following experiment on an ass about six months old, for the purpose of ascertaining the probable quantity of blood which an animal will lose before it dies.

"The weight of the animal being ascertained to be 79lbs. a puncture was made with a lancet into the jugular vein, from which the blood, which flowed in a very free stream, was collected. The vein having ceased to bleed, the carotid artery of the same side was divided, but no blood came from it: in a few seconds afterwards the animal was dead. The weight of the carcase was now found to be 73½lbs. consequently it had sustained a loss of 5½lbs. precisely the measure of the blood drawn. It appears from this experiment, that an animal will lose about 1-15th part of its weight of blood before it dies; though a less quantity may so far debilitate the vital powers, as to be, though less suddenly, equally fatal. In the human subject, the quantity of blood has been computed at about 1-8th part of the weight of the body; and as such an opinion has been broached from the results of experiments on quadrupeds, we may fairly take that to be about the proportion of it in the horse: so that, if we estimate the weight of a common sized horse at about 12cwt. the whole quantity of blood will amount to 84 qrts. or 168lbs. of which about 45 qrts. or 90lbs. will commonly flow from the jugular vein prior to death; though the loss of a much less quantity will deprive the animal of life.* 8.

It has been asserted that the horse's blood does not cup or buff—but this Mr. Percivall avers to be an error, as the phenomena are by no means unusual even when the horse is in perfect health. Of this he mentions a remarkable instance, which was noticed while making some experiments.

"A horse, to every appearance in perfect health, was bled to one pound; after which he was galloped (for the space of about twenty minutes) until he sweated profusely; while under extreme agitation, from the exertions he had been put to, another pint of blood was drawn, by unpinning the same orifice. The coagulum of the first parcel of blood was sizy, tough, contracted, and deeply cupped: that of the last exhibited no signs whatever of buff, was extremely loose and flabby in its texture, so that on being handled it readily

---

* "Supposing a man to weigh 12st. or 168lbs. the quantity of blood contained in his body may be rated at 21lbs. or 2 galls. 2 qrts. and 1 pint. Again, granting that a dog weighs 40lbs. the amount of his blood will be five pints. These calculations are useful, and worth our retention, inasmuch as they serve to guide us in practice, as to the probable extent to which we may, with safety, carry venesection in different animals. For instance, we may reckon the loss of a pint from a man to be equivalent to that of a gallon from a horse, or to four ounces from a dog, and *vice versâ;* selecting individuals from each class about the respective weights we have here set down."

mingled with the serum; and in a much shorter time than the first, went into the putrefactive state.

"This latter fact is intimately connected with what we have already advanced, regarding the non-coagulation of the blood after an animal has been coursed to death; since, had exertion been continued until the horse sunk under it, the blood would probably have remained wholly fluid; whereas, in this case, the animal being only in progress towards that state—being only urged to a point from which he could recover, the coagulating powers of the blood were merely diminished." 28.

2. *Pulse.* The pulse of a horse is about 45, and that of a dog 90 in the minute. The most frequent pulse which our author ever observed in a horse was 120. When the number of pulsations sinks much below the ratio abovementioned, it is generally an indication of disease in the brain, "and is very commonly met with in the lethargic or sleepy stage of staggers." The heart of the horse may generally be felt beating by applying the palm of the hand to his left side, immediately behind and a little above his elbow. In respect to the pulse, the best artery for feeling it in the horse is the submaxillary, as it crosses the posterior margin of the lower jaw. Digitalis, and white and black helebore, diminish the frequency of the horse's pulse. Aloes takes the appetite away and causes nausea also, but does not lessen the velocity of the pulse. Barytes given to the horse in large doses causes the pulse to intermit.

3. *Inflammation and its Consequences.* It is curious that no instance has been observed of *mortification*, as a termination of inflammation, in the horse.

"Horses who die of severe injuries, followed by high and extensive inflammation, sink prior to the gangrenous stage, and they appear to do so from excessive nervous irritation. The nearest approach to sphacelus, and one which we can adduce, if we can any, as an example of it, is that condition of the lungs where death has quickly supervened on acute inflammation of them: even here however, the parts cannot, correctly speaking, be called gangrenous." 69.

In horses, as in men, venesection, is the main remedy for inflammation. Thus, in what is vulgarly called "fever in the feet," or inflammation of the laminæ, we are to lose no time in opening the inferior coffin artery, and taking away three or four quarts of blood, which will generally arrest the disease in limine. So in staggers, we are to open the jugular veins, by which we have the advantage of both local and general blood-letting. Purging is of such extensive utility in veterinary practice that without it they could do nothing.

"Aloes, almost the only medicine on which we can depend as a purgative for horses,* is to be administered in doses apportioned to the size and apparent strength of constitution of our patient, and to the impression we wish to make on the system. To a horse in health, under ordinary stable management, we give about six drams;† to one with staggers, a disease in which there is much torpor of the bowels, we would give twelve: but the judgment of the practitioner will best determine the dose." 77.

Should the first dose not take effect it is seldom safe to repeat it in less than 24 hours—the period at which its operation commonly commences.

"Sometimes we exhibit aloes in a laxative form: with this view, we give two or three drams, or from that to half an ounce, once‡ a day, or every other day, until purging is produced. Now and then, an alterative medicine is compounded of it—the efficacy of which simply depends on a still smaller division of the aloes: we give one, and even half a dram, § once or twice a day, or every other day, according to the nature of the case." 77.

Veterinary surgeons bleed with good effect in the inflammatory diseases of horses, and also purge—but they are told that they cannot nauseate the animal with good effect. The horse's fauces are so constructed as plainly to demonstrate that it was not designed he should vomit like a man—and only one half of his stomach is susceptible of the operation of medicine.

"But, opposed to these facts, we have two others equally true, in practice;—the one is, that when we excite efforts to vomit, no ill effects whatever result; and the other, that the greatest benefit is derived from nausea, a state we can at any time produce and regulate with equal certitude. The root of white helebore|| is the medicine we have been in the habit of employing for this purpose for the last ten years, and we feel no scruple in adding, with the best effects;—it lowers and weakens the pulse, relieves the distended vessels of

---

* "We shall discuss this question at large, in our lecture *On Purgation, and Purgative Medicine.*

† "R. Aloës Vulgar. Ext. Pulv. ʒvj. vel. ℥i.

Ol. Menth. Piper. gtt. xx.

Syrupi—q. s. ut ft. Bol. statim sumend."

‡ "R. Pulv. Aloës. Vulg. ʒi. vel ʒij. vel. ʒiij.

Pulv. Glycyrrh Rad. ℥ss.

Pulv. Zingib. —— ʒi.

Syrupi ———— q. s. ut ft. Bol. omni die, vel om. alt. die sumend."

§ "Ut supra."

|| "Neither ipecacuanha, nor the tartarized antimony possesses virtue as a horse medicine. Very large doses of them may be given—two ounces of the latter, without producing any apparent effect."

the inflamed part, and promotes that smooth and glossy condition of skin, which is evidence sufficient, in the horse, of our having excited diaphoresis: these are not fabricated reports—they are the result of long experience and close observation, and such as we are not likely to concede to argument grounded upon any analogical or theoretical conclusions. The common dose is a scruple* of the powdered root, once, twice, or thrice a day, according to the effect we are desirous of producing: during its exhibition we are frequently to examine the pulse with attention, in order that we may detect its influence on it as early as possible, and either omit its subsequent use altogether, or give it in smaller doses, or at longer intervals. The effect of nausea is further indicated, by the horse's becoming suddenly dull and torpid—by his hanging his head down, or resting it upon the manger—by his refusing to eat, or drink—and by his frothing at the mouth: which last event is characteristic of excessive nausea, and only precedes, should the medicine be carried further, distressing and unavailing efforts to vomit. Even this effect of it, however, is attended with no danger, and only requires a little time to subside. On the whole, next to aloes, it is the best medicine with which we are acquainted to combat acute inflammatory disease; and the *only* one we are yet in possession of as a diaphoretic." 79.

Diuretic medicines are very generally given to horses, and are very serviceable in inflammatory affections. Common turpentine, resin, and soap, are the remedies chiefly employed, conjoined or simply mixed with any inert substance, to form what is called a urine or diuretic ball. They should be given every day, or every other day. But it must be remembered that the horse is very susceptible of diuretic medicines, and if they be given in too large doses, or too frequently repeated, they induce great debility, and sometimes nephritis. Narcotic medicines have not been used in the diseases of horses. With reference to diet and regimen, in inflammatory affections, it is proper to substitute bran for corn or beans—and to feed them with green meat, in lieu of hay, (in spring or summer,) unless the animal have inflamed bowels, when fermented provender will be best. When exercise is desirable, it should be confined to walking. There is no instance, Mr. P. observes, where a sick horse can be benefited by trotting or galloping—but he has seen many where simple disorder has been converted into alarming and fatal disease.

As a *topical* application in the inflammation of horses, nothing is better than the coldest water, or ice itself, inclosed in bags, and frequently renewed. Some people prefer the solu-

* " R. Pnlv. Rad. Veratri ℈j. vel. ℈ij.

Pulv. Flor. Anthemidis ℥ss.

Syrupi q. s. ut ft. Bol. bis terve die sumend."

tions of lead, with vinegar or spirits, to form an evaporating lotion. Fomentations are not very often necessary. The warm bath is only used in cases of strangles, where suppuration is to be promoted. Blisters, rowels, setons, rubefacients, sinapisms, and the actual cautery, are never, of course, to be employed when there is actual inflammation, but only to remove the effects or sequelæ of that disease.

"The actual cautery, (firing,) considered as the veterinary's last resource, is one of his most potent, and when properly handled, one of his most effectual means to renew inflammatory action, and excite absorption: it does the former by its immediate stimulating effects; the latter, it commences by stimulation, but promotes considerably afterwards by pressure, having the same effect as a tight bandage applied to the part." 84.

4. *Aneurism.* It is curious that, considering the violent exertions to which the horse is so frequently excited, there should yet be so few instances of aneurismal affections. Only one specimen is preserved in the Veterinary College, of aneurism of the thoracic aorta, about the size of a pumpkin.

5. *Veins.* There is but one disease of veins met with in the horse, and that the result of external injury. Ignorant farriers, however, describe "*blood-spavin*" as a varix of the horse's veins; but this, Mr. P. observes, is quite imaginary.

"If ever you examine a horse said to have blood-spavin, (for it is by no means a very common occurrence,) you will perceive a soft fluctuating tumor upon the inner and fore part of the hock, in the course of the principal vein, which is at that part superficially placed. At first view of it you are convinced from the unnatural prominence of the part, that there must be disease; and so there undoubtedly is; though it is not of that kind which its name so *emphatically* expresses. Dissection has fully developed its nature. There is placed here a little membranous bag, called a *bursa mucosa;* which contains in a natural state, a certain quantity of mucous fluid; from a too copious secretion of which, it happens, now and then, that this sac becomes distended, preternaturally enlarged, and in this condition constitutes a disease, called *bog-spavin;* the nature of which will be hereafter more fully explained. The vein, passing immediately over this bag, compressed, and diminished in calibre by enlargement of it, cannot transmit blood, at this part, with the usual facility or quickness; the consequence is, that a preternatural distention of it happens immediately below the tumid bursa, thence extending as low down as the first valve: and this has been mistaken for a varix, or some such thing, and denominated a *blood-spavin.*" 96.

Wounded veins, then, (from bleeding,) are the only diseases to be mentioned. If, from bad lancets, inattention in lying the vein, subsequent friction, or mal-habit of the animal, any separation of the sides of the wound takes place, tumefaction of the neck ensues. The horse should be tied up for an hour after bleeding, so that he cannot rub his neck; for some horses have bled to death by accidents of this kind. If inflammation of the vein take place, it is sometimes troublesome, and even dangerous, producing great constitutional irritation, denoted by quickened respiration, frequent and strong pulse, refusal of food, sparing evacuations of fæces and urine, and general heat of the body, especially of the mouth. In one case under Mr. P. death took place. It is not a little remarkable, that when the jugular vein of a horse inflames after bleeding, the disease spreads *against* the current of blood—that is, towards the head; whereas, in the human subject, the inflammation proceeds *with* the current of the circulation—towards the heart. Mr. Percivall's explanation not being very satisfactory, we shall pass it over.

"The mode of treatment found to be most successful by veterinary practitioners, is, in some respects, very different from what surgeons are in the habit of adopting. If the tumefaction and induration, in the course of the vein, have extended to the head, a blister is by far the best application, without any regard to fomentations, evaporating lotions, &c. which are only of use in the more incipient stages. At the same time, you should produce a slough, and excite the adhesive action in the vein, by the introduction of some caustic, or of the actual cautery; the latter is by far the preferable remedy. In the use of it we avoid all force—the mere searing of the sides of the wound is all that is required; our objects being to procure a free opening, and a discharge of healthy pus." 106.

Purgatives, in full doses, are proper; and the substitution of bran for corn is a good regimen. The head and neck should be kept as motionless as possible.

5. *Cellular Membrane*. Under this head we have the following useful direction, which should be attended to carefully, as we have seen fatal effects from neglect of the precept.

"Young horses recently brought up to London for sale, or others just taken from grass, or that have been fed on *soft meat* in the stable, and little exercised, are all fat and *fine to the eye*, but not *in condition for work*. The common and simple management that such horses require, to put them into condition for regular work, is to give them nothing but dried provender of the best kind; to exercise them gently at first, and exert them more by degrees; and occasionally administer moderate doses of aloes, by way of emptying the bowels, and exciting the powers of absorption, in order to get rid of some of their superabundant fat. By these means, we disencumber the muscles of their useless cellular and adipose matter, reduce the weight of the carcase, and improve the wind: in fact, we put the animal in a fit condition to go to hard work, or, in common language, *season him.*" 139.

6. *Neurotomy.* To Mr. Sewell, the able assistant professor at the Veterinary College, belongs the honour of first proposing or practising this now celebrated operation. If the withdrawal of pain not to be assuaged by other means—if the restoration of an incurably lame horse to a state of comparative soundness—be considerations of importance:—then neurotomy will take the lead of modern discoveries in veterinary science. Mr. Sewell has not escaped the envy that waits on all discoverers. First, the utility is denied—and, that ground no longer tenable, the originality, of course, is to be impugned.

The nerves being the media through which sensibility is conveyed—pain as well as pleasure—it is natural to expect that men should long ago have thought of interrupting the communication with a painful part by division of the nerve. Accordingly attempts, though fruitless ones, were made at the Veterinary College, by simple division of the nerve, many years ago, and the merit of discovery is accordingly denied to Mr. Sewell.

The division, or excision of the nerve in the pastern of a horse, does not appear to check the growth of the foot, or produce any disease thereof. If, then, the nerves of this organ be of so little apparent use, why is it so plentifully supplied with nerves?

"This operation has certainly taught us one, if not two important uses of nerves to the foot, which did not occur to us before: *viz.* that they (the nerves) are the safeguards of that organ in health, and, if we may be allowed the expression, its nurses in disease; they inform the animal when the foot is impaired, and warn him that it will be still farther injured by being made use of:—pain is the consequence of injury, the limb is favored, and the disease in it arrested simply from repose; so that it now and then subsides without medical aid. But in the insensible foot, the reverse of this is the case: the disease is aggravated by the animal's using the part as he would were it perfectly healthy; (he being totally unconscious of its presence;) and a destruction of the organization of the foot, if not the loss of life itself, may happen in consequence of a comparatively trifling accident." 173.

These considerations shew the necessity of great care and frequent inspection of the foot, whose nerves are cut. To the question—"do such horses go with that natural ease and freedom that others possess;" Mr. Percivall answers that he thinks and believes that the animal loses a something—probably a kind of feeling which affects his tread sufficient to inform his rider that his feet are not what they originally were.

The place for operating has now become a subject of dispute—some preferring the fetlock—some choosing to operate just above that part—while others cut the nerve on one side above or below the joint. "Mr. Sewell, and all who follow his directions, choose now the sides of the large pastern bone —*below the fetlock*, for the operation."

"Mr. Sewell's object in excising the large pastern nerve, is to preserve sensation around the front of the foot—a part, in general, free from disease; and this is now fulfilled, since the nerve that goes thither, is a branch from the metacarpal.

"The operation is by no means difficult to him acquainted with the anatomy of the part. The horse being cast, and properly secured, and the affected limb extended and placed in a convenient position for the operator, an incision, about $1\frac{1}{2}$ inch long, is to be made upon the side of the large pastern bone, opposite to, and in the direction of, the large pastern nerve; then, having cut through the cellular substance, so as to lay bare the trunk of this nerve, (anteriorly to which, and in contact with it, lies the artery, which must be carefully avoided,) raise it with a tenaculum, and divide it by the introduction of a probe-pointed bistoury, as high up as the external wound will admit: lastly, clean it from surrounding adhesions, and detach it from the continuation of the trunk below, so as to excise a portion about 3-fourths of an inch in length. The operation requires to be performed on each side, there being two separate trunks below the fetlock; and, of course, on both legs, should both be affected. To close the wound, we rather employ adhesive plaster than suture, with straps of which the pastern may be encircled, if the hair be closely shorn off: where suture is preferred, the interrupted is the best, and about three or four stitches are sufficient. The legs should be bandaged* after the operation, and the horse not allowed to lie down during the first night, or be moved for the three or four following days, lest the parts be disturbed, and union by adhesion defeated." 176.

Mr. Sewell has operated on upwards of 500 horses, and in eight cases out of ten, with full and complete success. For want of proper management subsequently to the operation, some few have turned out unfortunately. It would appear, from the reports transmitted to Mr. Sewell, from various quarters, that two years or thereabouts may be calculated as the term which a horse will run in hard labour, after this

* "We commonly dip the bandages, previously to applying them, in the saturn wash. It is also a good practice, to give a dose of physic, and keep the horse, during his confinement, on bran, instead of corn. We may observe, that, in many cases, the bandages only are sufficient."

operation, when successful. It is curious that two instances have occurred to Mr. Sewell, one in a horse and another in a mare, where the copulative functions returned after neurotomy, though they had ceased previously from, it is supposed, the pain of the foot. Our readers are aware that Mr. Swan, of Lincoln, performed this operation on the human subject. The great difficulty lies in selecting proper objects for the operation. The following are our author's rules for our guidance on this point.

" 1*st*. Any kind of *chronic* lameness about the foot, or coronet, with the exception of that arising from pumice-feet, may require the operation.

" 2*nd*. Should inflammation be present in the foot, pasterns, or fetlock, it is always to be reduced prior to the operation.

" 3*rd*. If we suspect ulceration of the joints—of the ligaments, tendons, or articular cartilages, we should not operate; since neurotomy will aggravate the disease, by enabling the animal to make use of parts, unfit, in their present condition, to bear either pressure or motion.

" 4*th*. Neurotomy succeeds best in those cases in which lameness is accompanied by an alteration in the form and texture of the hoof, (pumice-foot excepted,) and where all previous disease has terminated in this: indeed, to some horses lame from anchylosis, partial or complete, it seems often to convey permanent benefit, by inducing them to exert these parts more, and thereby obtaining, in process of time, some small degree of useful motion in joints before stiff and immoveable." 184.

Mr. Sewell finds that, in cases of entire section of the nerve, sensation returns in about two months. Where excision of a portion of nerve takes place, the period of returning sensation is very uncertain. In one of Mr. Sewell's own horses no feeling had returned at the end of three years. It is undecided whether lameness is likely to return with sensation.

Tetanus is not a very rare disease of horses, and is nearly as fatal in those animals as in the human subject. Venesection produces more relief than any other measure, but this and every other remedial agent too often fail.

7. *Tendons.* The tendons of the flexor muscles of the fore legs are most frequently the seat of injury or disease in the horse.

" What is called a sprain, or strain of the flexor tendons of the leg, does not, in the generality of cases, consist in any preternatural extension of those parts, but in a laceration of that cellular membrane which connects them together, and of which the thecæ for them is composed, in their passage between the knee and fetlock-joint. We do not mean to contend, that in the worst accidents of this kind the tendons may not be stretched, and even partially ruptured; but we would reject the accounts of writers altogether, in reference to their being *torn asunder*, as wholly unsupported by the actual inspection of these parts, and thus far disbelieve that a horse was ever *broken down*." 203.

On the treatment of this very common accident we shall give Mr. Percivall's directions in his own words.

"The treatment of a slight sprain is very simple. If the leg be but little swollen, and the lameness inconsiderable, the use of some refrigerent, or evaporating lotion,* with a well applied bandage, will be all that is required; or—a practice some prefer, immerse the leg in spring water, and let the horse stand up to the knee in it for several hours in the day, using the bandage and cold wash only during the night. When there is much heat, swelling, and tension of the leg, however, our treatment of the case must be more active. In the first place, we are to draw blood to the amount of from four to six pounds; and this we can always do, with as much effect as locally, by removing the horn at the toe and puncturing the anterior coffin artery, or opening the cephalic, or saphena vein, according as it is the fore or hind leg affected. We should also, at this time, exhibit a full dose of purgative medicine.† Emollient applications are here to be preferred to cold; fomenting the leg with warm water, or, what is more effectual, steeping it in the same manner as that in which we have recommended the use of the cold, will tend much to the reduction of the inflammation, and considerably alleviate the animal's sufferings. When the heat and tenderness have much abated, we prefer immersion in cold to warm water, or have recourse to the evaporating lotion and bandage; taking care, however, not to apply the bandage tightly, until the animal can bear moderate compression of the leg with the fingers. Rest is indispensable: it is an erroneous practice to turn horses loose into stables at the onset of this disease; though it is of great service to them when the inflammatory action has subsided; which you may determine on by the feel, and by the animal's *walking* sound." 205.

The swellings and stiffnesses that frequently remain after the inflammation has disappeared must be treated on the same principles that guide the medical practitioner when man is the subject.

8. *Muscles.* Under this head we shall only glance at *Stringhalt,* a convulsive movement of the hinder extremities

---

* "Either the Liquor. Plum. Subacet. Dilut. or that in combination with Vinegar, or Spirits of Wine.—Vide Lecture V. *Local Treatment of Inflammation.*"

† "R. Aloes Vulgar. Ext. ʒvij.

Ol. Carui gtt. xl.

Syrupi q. s. ft. Bol. for a horse of ordinary size."

of the horse, corresponding, in some slight degree, with chorea in the human subject. "Not unfrequently when the animal has lifted his hind leg from the ground, which is always done with a convulsive twitch, the fetlock nearly approaches the belly, and by some other remarkable irregularities in its action, before the foot can be replaced upon the ground, displays such unnatural movements as to convince us that volition has little power over it during its suspension." It is said that in many parts of the Continent this equestrian chorea is esteemed extremely graceful—particularly if it affects both legs! It is considered by our author as the consequence of some affection of the spinal marrow or nerves proceeding thence.

9. *Bones.* No animal is more subject to exostosis than the horse, and it is a very common cause of permanent lameness. The pathology of our author, here, as on all other occasions, is taken from medical writers, and therefore we do not touch upon it. The best remedy is constant blistering—and then the actual cautery. At the veterinary college it is the practice to divide the periosteum in splints.

"We would define a spavin, (called by horse-dealers and grooms a *jack*,) to be an exostosis upon, or near to, the inner and lower part of the hock. In its origin and progress it is very similar to a splint; indeed, it may be, in reality, purely a splint; although from its situation, we should denominate it a spavin. To explain this, a spavin may, and commonly we believe does, arise from an inflammation of the cartilago-ligamentous substance connecting the head of the inner small metatarsal to that of the cannon bone, without any accompanying disease of the bones of the hock; and this, terminating in ossification, may be a splint as to its nature, but is a spavin as to its situation." 352.

We must here close our short notice of the work before us. On perusing it attentively we cannot recommend the purchase of it by our professional brethren—and for this reason only, that nineteen-twentieths of it are taken from the best medical and surgical writings, which writings are familiarly known. In short, we have given almost the whole of what really appertains to the *veterinary* art. At the same time we can have no doubt but that the book is very well adapted to the veterinary student—and that it is the most scientific, as far as it yet goes, of any work emanating from a similar source.

